# The response of the Women’s Dermatologic Society and the *International Journal of Women’s Dermatology* to the COVID-19 pandemic

**DOI:** 10.1016/j.ijwd.2020.04.008

**Published:** 2020-05-04

**Authors:** Jenny E. Murase, Dedee F. Murrell

**Affiliations:** a Department of Dermatology, UCSF, San Francisco, CA, United States; b Palo Alto Foundation Medical Group, Mountain View, CA, United States; c Department of Dermatology at St. George Hospital, University of New South Wales, Sydney, Australia

The Women’s Dermatologic Society (WDS)’s vision statement reinforces the commitment of the Society to issues relevant to women and their families. During this time of the COVID-19 pandemic, the WDS is working to mitigate the challenges caused by the pandemic and support dermatologists in our community. As stated in the COVID-19 Response Statement from President Molly Hinshaw, MD: “You will see greater attention to remote learning, mentorship and networking opportunities across our programs. We are working…with our industry partners on novel educational initiatives.”

As co-editors-in-chief of the journal of the WDS, the *International Journal of Women’s Dermatology*, we feel it is of paramount importance to publish information relevant to physicians, women, and their families during the COVID-19 pandemic. To this end, we have included five signature pieces in the June 2020 issue that are directly relevant to the pandemic. Our Open Access allows all individuals the ability to access these informative publications, thereby providing information that is peer reviewed and reliable to the public at a time when information that is not medically accurate regarding the pandemic is pervasive on the Internet.

In the Art of Prevention series article by senior author Dr. Sharon Jacobs (founder of the AOP series) and colleagues, entitled “Life in the time of coronavirus” (Baghchechi et al., 2020), precautions and recommendations to prevent coronavirus disease spread and risk assessment questions for providers to ask patients to assess disease risk are outlined for health care providers and the community. The epidemiology of the viral spread and patient presenting signs and symptoms are clarified, and a list of practical intervention pearls to prevent disease transmission are described in detail.

In the review article “Management guidelines for pregnant healthcare workers exposed to infectious dermatoses” (Reddy et al., 2020) that I co-authored with Vidhatha Reddy, Alexander Kollhoff, and Kathryn Martires, we discuss the management recommendations for pregnant health care workers who could be providing clinical care for patients exposed to COVID-19. We also discuss recommendations for these workers who develop symptoms of COVID-19 as well as guidelines for the management of any pregnant health care worker who tests positive for the virus.

In the editorial “The role of virtual support groups for hidradenitis patients during the COVID-19 pandemic” (Stout, 2020), Dr. Stout introduces the novel concept of developing virtual patient support groups for chronic dermatologic diseases to provide much needed emotional support during this time of physical isolation and social distancing. Dr. Stout provides an example of a virtual hidradenitis support group that her research group created and offers an example of the process used by the physicians that could potentially be used by others to create support groups for a variety of chronic and debilitating dermatologic conditions during the pandemic.

In the editorial “The dermatologist’s perspective: Why is COVID-19 mortality lower in females than males?” (Murrell and Murase, 2020), as co-editors-in-chief we reflect on why epidemiologic statistics reveal almost double the mortality in men versus women during this pandemic. We comment on potential behavioral and medication changes that may help reduce the risk.

Finally, in the ethical analysis “Dermatoethics: Self-prescribing plaquenil during the COVID-19 pandemic” (Stoj and Grant-Kels, 2020), founding co-editor-in-chief of the *International Journal of Women’s Dermatology* Dr. Jane Grant-Kels discusses the ethics involved when contemplating prescribing hydroxychloroquine (plaquenil) so that a provider could personally have the medication on hand during the pandemic if family members become ill from the virus. She reflects on how the actions of dermatologists could be considered “unethical and an assault on distributive justice” should a shortage be created so that others in the community who need this medication for their illnesses no longer are able to access it due to stockpiling for personal emergency.

We believe that publishing these pieces will provide information to dermatologists and health care providers around the world so that they may arm and protect their patients as well as themselves, particularly should they be exposed to the virus during pregnancy. We hope that physicians and practitioners are inspired to use technology to reinforce connections in our virtual world and support patients with chronic, debilitating dermatologic disease who are suffering as a result of the social isolation of their disease itself, made worse by governmental shelter-in-place edicts and social distancing. Finally, we hope that it provides an avenue for thoughtful reflection on how our actions affect those around us and that it reinforces a concept that we have only recently come to realize more so than we have in the past: that we are ultimately connected in ways that we had not realized prior to the COVID-19 pandemic, and our actions have far-reaching effects on many others whom we may never know.
